# Complete Surgical Resection of a Giant Invasive Thymoma with Right Pneumonectomy and Graft Reconstruction of the Superior Vena Cava and Left Brachiocephalic Vein: A Case Report

**DOI:** 10.1155/2022/9604926

**Published:** 2022-11-28

**Authors:** Savvas Lampridis, Rajdeep Bilkhu, Gianluca Lucchese, Andrea Billè

**Affiliations:** ^1^Department of Thoracic Surgery, Guy's Hospital, London, UK; ^2^Department of Cardiac Surgery, St Thomas' Hospital, London, UK

## Abstract

**Background:**

Complete surgical resection represents one of the most important prognostic factors for thymomas. However, surgery is usually not considered when there is invasion of the pulmonary hilum and mediastinal veins because of technical considerations or potential perioperative morbidity and mortality. *Case Presentation*. We present the case of a 37-year-old woman with a giant thymoma infiltrating the superior vena cava, left brachiocephalic vein, and most of the right lung. Following 3 cycles of chemotherapy with minimal tumour response, she was hospitalised with COVID-19 and refused further systemic treatment. She subsequently underwent surgery after a thorough preoperative evaluation. Surgical resection of the tumour was performed with concomitant right pneumonectomy and reconstruction of the superior vena cava and left brachiocephalic vein using expanded-polytetrafluoroethylene grafts through a median sternotomy combined with a clamshell incision. Histopathological analysis of the resected specimens demonstrated a type B2, Masaoka-Koga stage IVa thymoma that was completely resected. Following an uneventful course, she was discharged home on the ninth postoperative day with anticoagulation therapy. She has remained free of disease or adverse events after a 12-month follow-up.

**Conclusions:**

Complete surgical resection of invasive thymomas with concomitant pneumonectomy and venous graft reconstruction is a feasible and safe procedure. Careful patient selection and adequate postoperative anticoagulation are crucial for successful clinical outcomes.

## 1. Introduction

The treatment of thymomas is primarily surgical, with completeness of resection representing a validated prognostic factor for disease recurrence and patient survival [1]. However, most patients with tumours invading the great vessels are not offered surgical resection due to technical considerations or the potential perioperative morbidity and mortality [2]. Additional technical challenges and increased surgical risks are posed by large thymomas that also involve the pulmonary hilum, thereby impairing visualization and control required for anatomic lung resection. Here, we report the case of a patient with an exceedingly bulky thymoma invading the superior vena cava (SVC), left brachiocephalic vein, and most of the right lung, which was treated with radical surgical resection. The case is presented in accordance with the CARE reporting checklist.

## 2. Case Presentation

**Figure 1:**
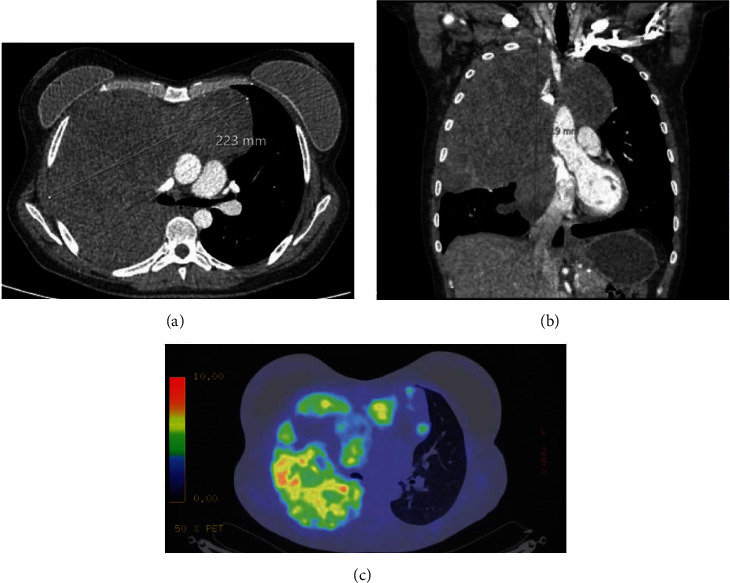
(a) Axial and (b) coronal computed tomography of the thorax showing a tumour invading the right lung, occupying most of the right hemithorax, and crossing the midline to the left side of the mediastinum. (c) ^18^F-fluorodeoxyglucose positron emission tomography integrated with computed tomography showing heterogeneous radiotracer uptake in the tumour, with maximum standardized uptake value of 13.1 and areas of photopenia.

A 37-year-old woman, without any significant past medical history, presented with neck muscle stiffness and underwent magnetic resonance imaging, which revealed an abnormality in the superior mediastinum. This finding was further investigated with thoracic computed tomography (CT), which demonstrated a lobulated, heterogeneous mass measuring approximately 22.5 cm in craniocaudal dimension, occupying most of the right hemithorax and crossing the midline to the left side of the mediastinum (Figures 1(a) - 1(b)). The tumour appeared to infiltrate the SVC, the left brachiocephalic vein, the upper and middle lobes of the right lung entirely, and the lower lobe partially, as well as the extrapericardial right main pulmonary artery and right inferior pulmonary vein. Percutaneous core-needle biopsy of the mass revealed thymoma. A subsequent ^18^F-fluorodeoxyglucose positron emission tomography (PET) integrated with CT did not show evidence of metabolically active lymph nodes, separate foci of radiotracer uptake in the pleura or pericardium, or extrathoracic disease (Figure 1(c)). The clinical TNM classification of the thymoma was cT3N0M0 according to the 8th edition of the TNM classification of malignant tumours published by the Union for International Cancer Control. Physical examination and serologic studies did not reveal evidence of myasthenia gravis or other paraneoplastic syndromes.

The initial treatment plan proposed by a multidisciplinary team included 6 cycles of induction chemotherapy, followed by restaging of the disease and further management depending on the results. The patient received 3 cycles of cyclophosphamide, doxorubicin, and cisplatin, but she refused further chemotherapy after she was hospitalised due to COVID-19. PET-CT demonstrated shrinkage of the area with the highest metabolic activity but overall unchanged dimensions of the tumour and invasion of adjacent structures. She was offered surgery with the potential risk of residual disease. Preoperative investigations showed forced expiratory volume in 1 second of 73% of predicted, diffusing capacity for carbon monoxide of 64% of predicted, and maximal oxygen consumption of 76% of predicted. Ventilation/perfusion scintigraphy revealed that the right lung contributed 29% to the global ventilation, and transthoracic echocardiography demonstrated left ventricular ejection fraction of 57% and normal pulmonary arterial pressures.

**Figure 2:**
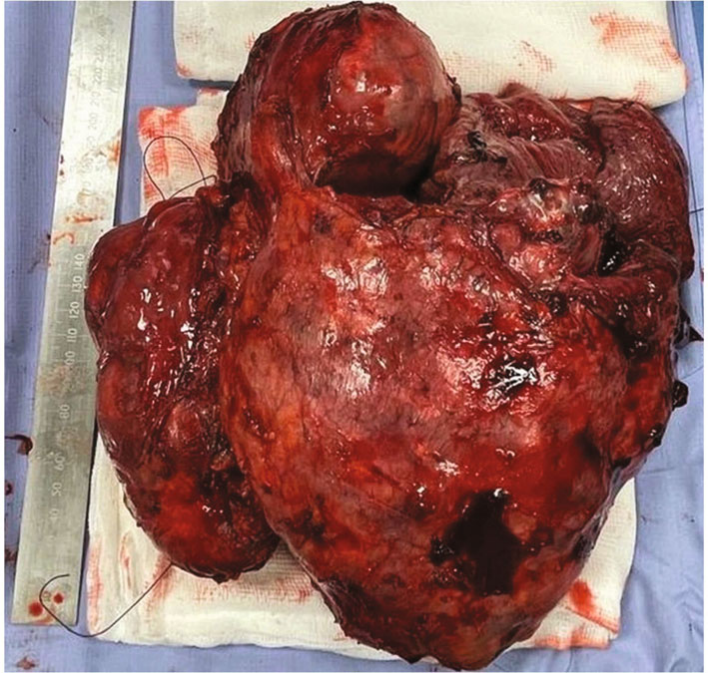
Giant thymoma resected en bloc with the right lung and ipsilateral parietal pleura. The specimen measured 236 mm × 214 mm × 114 mm.

**Figure 3:**
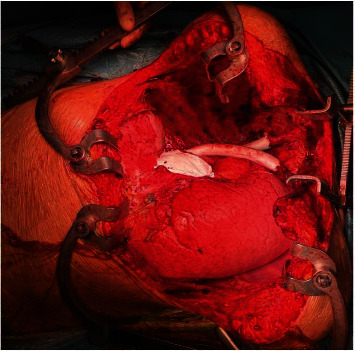
Intraoperative image following resection of an invasive thymoma with right extrapleural pneumonectomy, reconstruction of the superior vena cava and left brachiocephalic vein with expanded-polytetrafluoroethylene grafts, and replacement of the pericardium with an expanded-polytetrafluoroethylene mesh.

Surgery took place 3 months after the last cycle of chemotherapy, with the availability of cardiopulmonary bypass machine and a perfusionist in case there was involvement of the heart, aorta, or arch vessels. The patient was placed in the supine position and underwent general anaesthesia with double-lumen tracheal intubation and insertion of nasogastric tube, central and peripheral intravenous catheters, and radial arterial line. A median sternotomy and clamshell incision through the fifth intercostal space were performed. The tumour was initially dissected off the aortic arch, arch vessels, left main pulmonary artery, and left phrenic nerve, which was preserved. The pericardium was excised, and the tumour was dissected on the right side from the spine, oesophagus, and brachial plexus. A right exrapleural pneumonectomy was performed, and the thymoma was removed en bloc with the lung (Figure 2). The patient received intravenous heparin sodium (80 IU/kg) 10 minutes before vascular clamping. The left brachiocephalic vein and part of the SVC that was infiltrated by the tumour were resected. The SVC was reconstructed with a 10 mm expanded-polytetrafluoroethylene (ePTFE; Gore-Tex, W. L. Gore & Associates, Newark, DE, USA) interposition graft, and the left brachiocephalic vein was anastomosed to the right atrial appendage using the same synthetic conduit. The pericardium was replaced with a Gore-Tex (W. L. Gore & Associates) mesh (Figure 3). Further resection margins were obtained where the tumour was closely adherent to adjacent structures, ensuring complete macroscopic resection. Three chest drains were inserted, and the incisions were closed in a standard fashion. The partial thromboplastin time was allowed to normalize without pharmacologic reversal.

Postoperatively, the patient was transferred to the intensive care unit for close monitoring and subsequently to the ward (level 1 of care). To prevent graft occlusion, she was commenced on warfarin with a target international normalized ratio (INR) of 2.5-3.5. She was discharged on the ninth postoperative day, when her INR was within the target range, after an uncomplicated course.

Histopathological analysis of the resected specimens revealed a type B2 thymoma that measured 23.6 cm in maximal dimension and invaded the right lung, SVC, and left brachiocephalic vein. The tumour was characterised by extensive necrosis, hyalinisation, histiocytic reaction, and cystic and haemorrhagic degeneration, consistent with therapy changes and infarction, with an estimated 15% remaining viable tumour. Microscopic examination also revealed a separate tumour nodule in the pleura measuring 5 mm. The pathological TNM classification of the disease was ypT3N0M1a (stage IVA), and the Masaoka-Koga stage was IVa. Residual tumour posttreatment was classified as R0 (i.e., no residual tumour), considering complete resection of the thymoma, its locoregional extent, and distant metastasis. The case was rediscussed by a multidisciplinary team, with a consensus for surveillance only. The patient has been regularly followed up for approximately 12 months with physical examination and imaging investigations (chest radiography and contrast-enhanced CT), without evidence of disease recurrence, graft thrombosis, or other adverse effects. She has returned to her predisease performance and physical activities.

## 3. Discussion

The present case demonstrates that aggressive surgical management of locally invasive thymomas with concomitant anatomic lung resection and vascular reconstruction is feasible and safe in selected patients. SVC and brachiocephalic vein reconstructions were performed with ringed ePTFE grafts using the technique originally described by Dartevelle et al. [3] in 1986. Since then, however, the published experience has remained limited to a small number of case reports and series. As an example, in a study of 14 patients with invasive thymic malignancy undergoing reconstruction of the left brachiocephalic vein (*n* = 7) or the SVC and left brachiocephalic vein (*n* = 7) using PTFE or ePTFE grafts, no major complications or occlusive symptoms were observed postoperatively, except in one patient who died due to gastrointestinal haemorrhage [4]. In a more recent study, Sun et al. [5] reported 25 cases of thymoma invading neighbouring great vessels. Of those, 20 patients underwent complete resection of the tumour along with vessel reconstruction using PTFE grafts. The performed reconstructions included anastomosis between brachiocephalic vein and right atrial appendage (*n* = 11), SVC replacement (*n* = 8), or both (*n* = 1). Postoperative complications included pneumonia (*n* = 3), haemothorax (*n* = 1), chylothorax (*n* = 1), and atelectasis (*n* = 1), while 2 patients died because of acute respiratory distress syndrome within 90 days after surgery.

The number of published cases reporting vein reconstruction with simultaneous anatomic lung resection is very small. In such cases, a thorough preoperative evaluation to ensure adequate cardiorespiratory reserve is paramount for successful surgical outcomes. Furthermore, to facilitate exposure and control of the hilar bronchovascular structures, these patients may require an additional surgical approach, including hemiclamshell or clamshell incision. In our case, we chose to associate the median sternotomy with a clamshell incision due to the extension of the tumour in both pleural cavities. Indeed, a clamshell incision provides excellent exposure of the mediastinum and hemithoraces; however, it is a rather invasive approach and should be considered only when other incisions cannot provide adequate exposure.

Postoperative anticoagulation in cases of vascular reconstruction is important to minimize the risk of thromboembolic events. In a study of 16 patients who underwent resection of malignant tumours with a prosthetic interposition graft of brachiocephalic veins or SVC, 13 patients were discharged with warfarin therapy, and most of them received it for at least 6 months [6]. Of the 20 implanted grafts, 8 were occluded (7 of which were made of PTFE) at a median follow-up of 42 months (range, 1–107 months). However, in another study of 28 patients undergoing replacement of the SVC with PTFE graft for malignant tumours, venous thrombosis occurred only in 1 case within 4 months after surgery [7]. In the present case, we chose administration of warfarin with a relatively high INR target because of the increased risk of thrombosis and low risk of bleeding.

## 4. Conclusions

Complete surgical resection of invasive thymomas with concomitant anatomic lung resection and venous graft reconstruction is a feasible and safe procedure. Careful patient selection and adequate postoperative anticoagulation are crucial for successful surgical outcomes.

## 5. Patient Perspective

The patient kindly shared her thoughts on the treatment she received. She was overwhelmed when she heard about the diagnosis and extent of her disease. After she started chemotherapy, she believed that there was not much progress and felt hopeless. This is the reason she wanted to have surgery, despite her worry that there may be residual disease, as she was informed by her primary surgeon. She was subsequently extremely relieved when she found out that all her disease was removed, and she was also pleasantly surprised with how fast she went home and recovered from her surgery.
